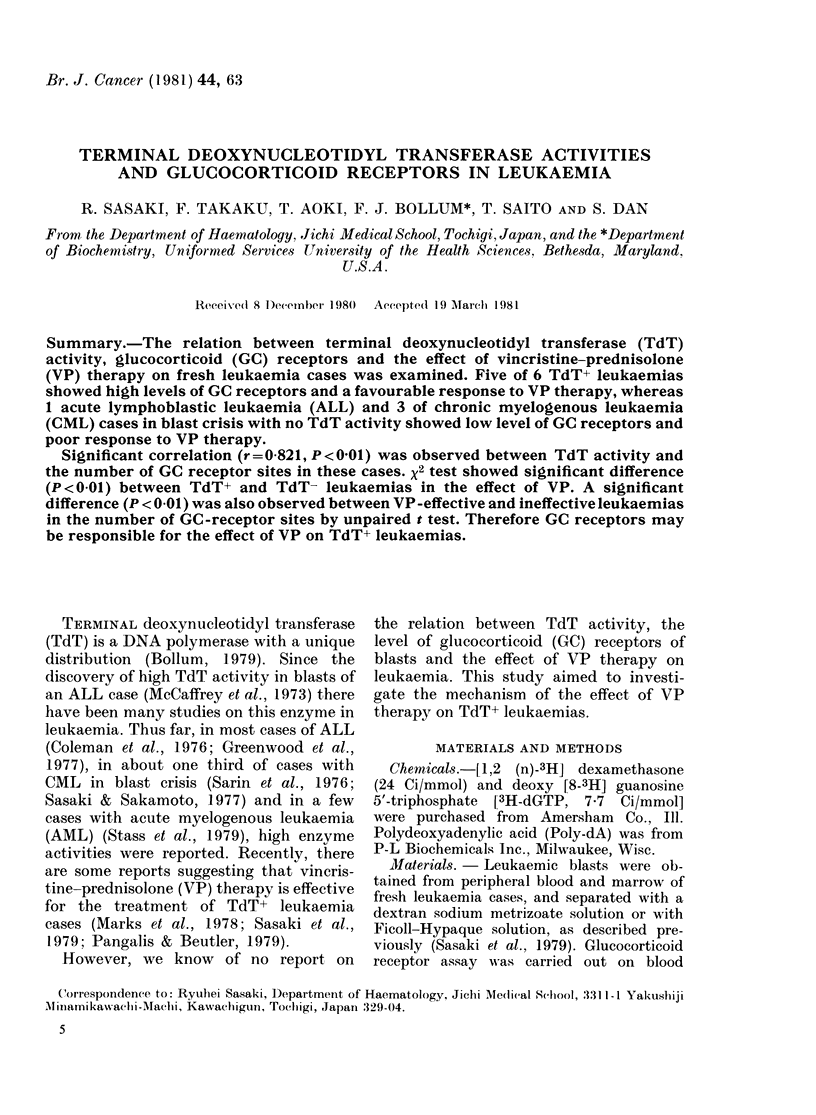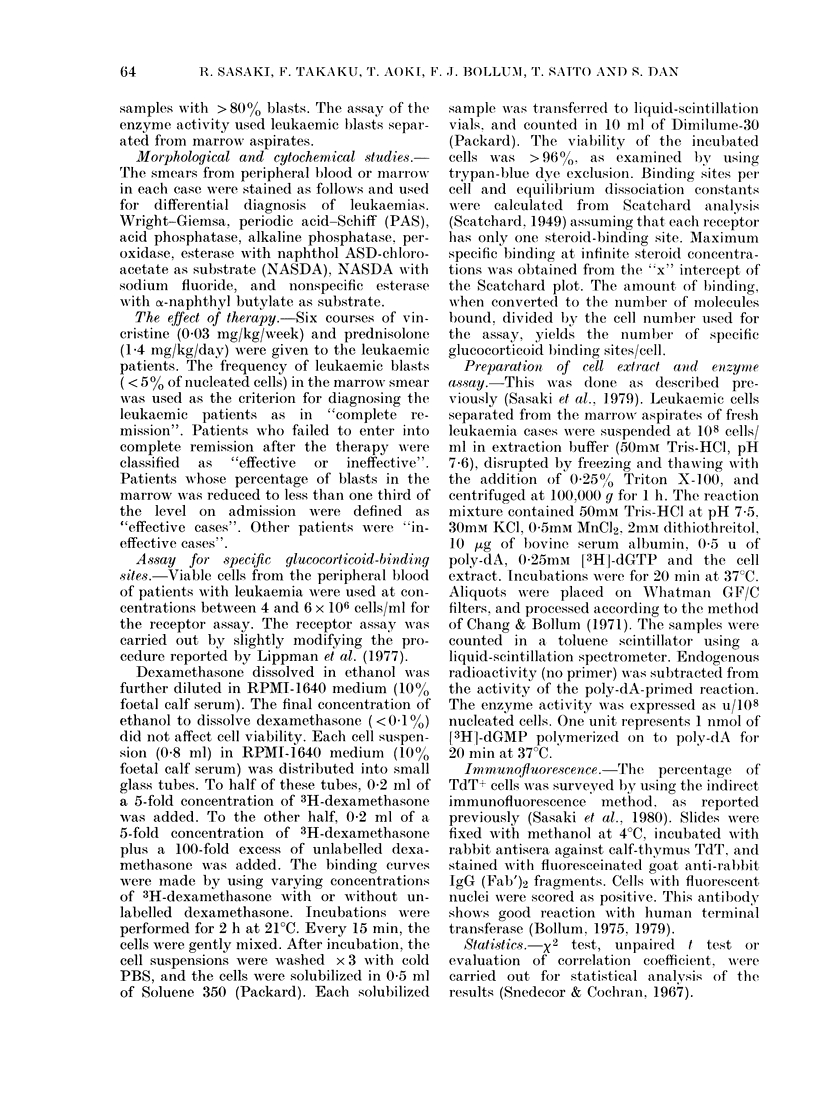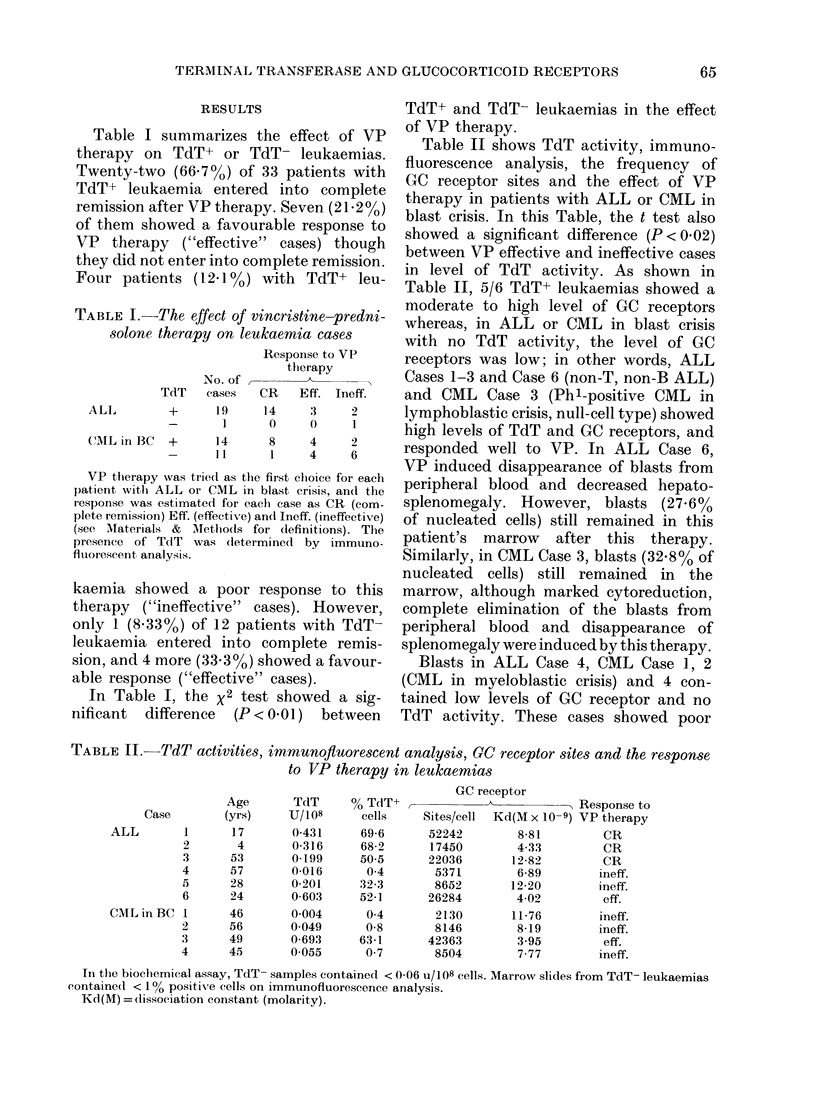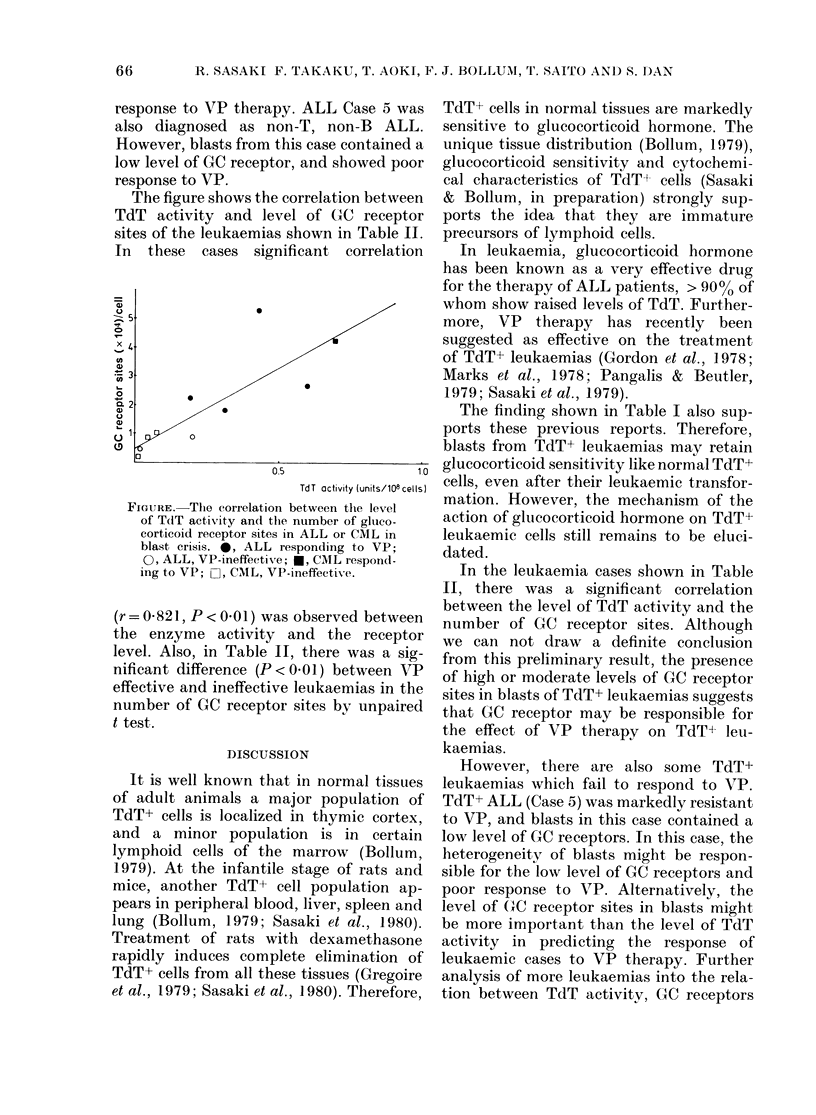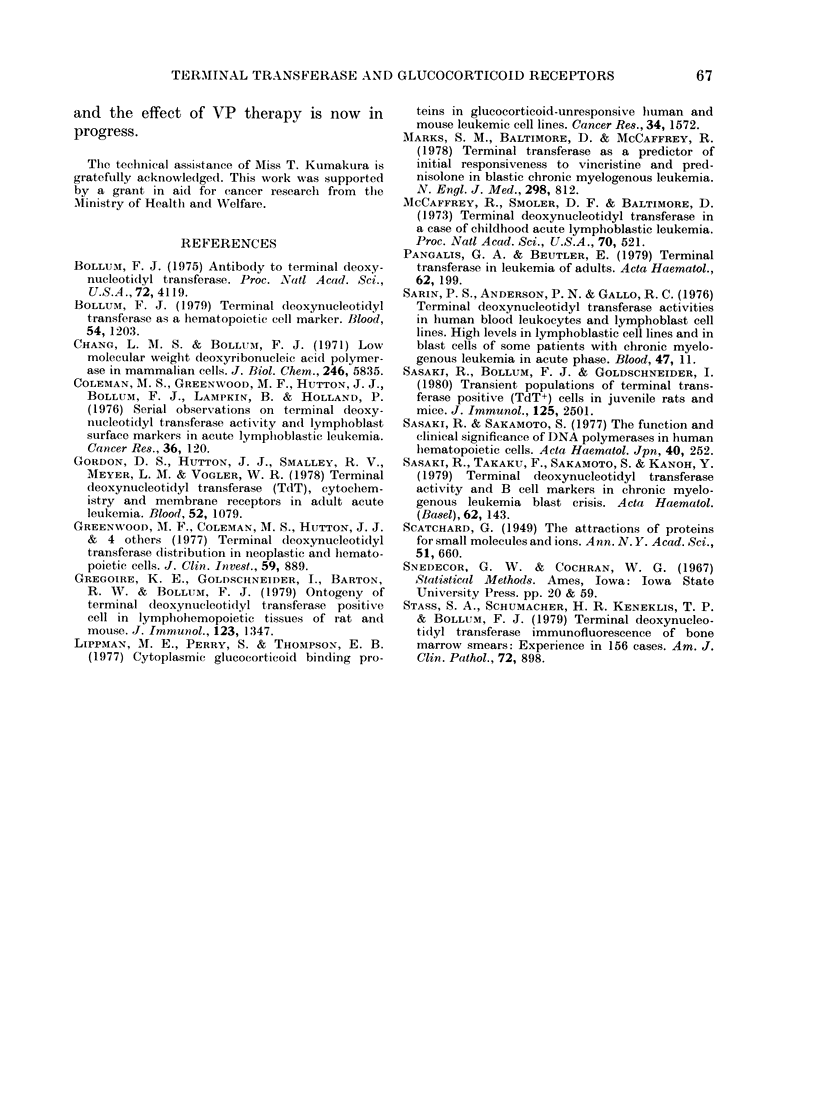# Terminal deoxynucleotidyl transferase activities and glucocorticoid receptors in leukaemia.

**DOI:** 10.1038/bjc.1981.148

**Published:** 1981-07

**Authors:** R. Sasaki, F. Takaku, T. Aoki, F. J. Bollum, T. Saito, S. Dan

## Abstract

The relation between terminal deoxynucleotidyl transferase (TdT) activity, glucocorticoid (GC) receptors and the effect of vincristine-prednisolone (VP) therapy on fresh leukaemia cases was examined. Five of 6 TdT+ leukaemias showed high levels of GC receptors and a favourable response to VP therapy, whereas 1 acute lymphoblastic leukaemia (ALL) and 3 of chronic myelogenous leukaemia (CML) cases in blast crisis with no TdT activity showed low level of GC receptors and poor response to VP therapy. Significant correlation (r = 0.821, P less than 0.01) was observed between TdT activity and the number of GC receptor sites in these cases. X2 test showed significant difference (P less than 0.01) between TdT+ and TdT- leukaemias in the effect of VP. A significant difference (P less than 0.01) was also observed between VP-effective and ineffective leukaemias in the number of GC-receptor sites by unpaired t test. Therefore GC receptors may be responsible for the effect of VP on TdT+ leukaemias.


*Br. J. Cancer* (1981) **44**, 63

### TERMINAL DEOXYNUCLEOTIDYL TRANSFERASE ACTIVITIES AND GLUCOCORTICOID RECEPTORS IN LEUKAEMIA

R. SASAKI, F. TAKAKU, T. AOKI, F. J. BOLLUM*, T. SAITO AND S. DAN

*From the Department of Haematology, Jichi Medical School, Tochigi, Japan, and the *Department of Biochemistry, Uniformed Services University of the Health Sciences, Bethesda, Maryland, U.S.A.*

Received 8 December 1980 Accepted 19 March 1981

**Summary.—The relation between terminal deoxynucleotidyl transferase (TdT) activity, glucocorticoid (GC) receptors and the effect of vincristine–prednisolone (VP) therapy on fresh leukaemia cases was examined. Five of 6 $TdT^+$ leukaemias showed high levels of GC receptors and a favourable response to VP therapy, whereas 1 acute lymphoblastic leukaemia (ALL) and 3 of chronic myelogenous leukaemia (CML) cases in blast crisis with no TdT activity showed low level of GC receptors and poor response to VP therapy.**

**Significant correlation ($r=0{\cdot}821$, $P<0{\cdot}01$) was observed between TdT activity and the number of GC receptor sites in these cases. $\chi^2$ test showed significant difference ($P<0{\cdot}01$) between $TdT^+$ and $TdT^-$ leukaemias in the effect of VP. A significant difference ($P<0{\cdot}01$) was also observed between VP-effective and ineffective leukaemias in the number of GC-receptor sites by unpaired $t$ test. Therefore GC receptors may be responsible for the effect of VP on $TdT^+$ leukaemias.**

TERMINAL deoxynucleotidyl transferase (TdT) is a DNA polymerase with a unique distribution (Bollum, 1979). Since the discovery of high TdT activity in blasts of an ALL case (McCaffrey *et al.*, 1973) there have been many studies on this enzyme in leukaemia. Thus far, in most cases of ALL (Coleman *et al.*, 1976; Greenwood *et al.*, 1977), in about one third of cases with CML in blast crisis (Sarin *et al.*, 1976; Sasaki & Sakamoto, 1977) and in a few cases with acute myelogenous leukaemia (AML) (Stass *et al.*, 1979), high enzyme activities were reported. Recently, there are some reports suggesting that vincristine–prednisolone (VP) therapy is effective for the treatment of $TdT^+$ leukaemia cases (Marks *et al.*, 1978; Sasaki *et al.*, 1979; Pangalis & Beutler, 1979).

However, we know of no report on the relation between TdT activity, the level of glucocorticoid (GC) receptors of blasts and the effect of VP therapy on leukaemia. This study aimed to investigate the mechanism of the effect of VP therapy on $TdT^+$ leukaemias.

### MATERIALS AND METHODS

*Chemicals.*—[1,2 (n)-$^3$H] dexamethasone (24 Ci/mmol) and deoxy [8-$^3$H] guanosine 5′-triphosphate [$^3$H-dGTP, 7·7 Ci/mmol] were purchased from Amersham Co., Ill. Polydeoxyadenylic acid (Poly-dA) was from P-L Biochemicals Inc., Milwaukee, Wisc.

*Materials.* — Leukaemic blasts were obtained from peripheral blood and marrow of fresh leukaemia cases, and separated with a dextran sodium metrizoate solution or with Ficoll–Hypaque solution, as described previously (Sasaki *et al.*, 1979). Glucocorticoid receptor assay was carried out on blood

Correspondence to: Ryuhei Sasaki, Department of Haematology, Jichi Medical School, 3311-1 Yakushiji Minamikawachi-Machi, Kawachigun, Tochigi, Japan 329-04.

samples with $>80\%$ blasts. The assay of the enzyme activity used leukaemic blasts separated from marrow aspirates.

*Morphological and cytochemical studies.*—The smears from peripheral blood or marrow in each case were stained as follows and used for differential diagnosis of leukaemias. Wright–Giemsa, periodic acid–Schiff (PAS), acid phosphatase, alkaline phosphatase, peroxidase, esterase with naphthol ASD-chloroacetate as substrate (NASDA), NASDA with sodium fluoride, and nonspecific esterase with $\alpha$-naphthyl butylate as substrate.

*The effect of therapy.*—Six courses of vincristine (0·03 mg/kg/week) and prednisolone (1·4 mg/kg/day) were given to the leukaemic patients. The frequency of leukaemic blasts ($<5\%$ of nucleated cells) in the marrow smear was used as the criterion for diagnosing the leukaemic patients as in "complete remission". Patients who failed to enter into complete remission after the therapy were classified as "effective or ineffective". Patients whose percentage of blasts in the marrow was reduced to less than one third of the level on admission were defined as "effective cases". Other patients were "ineffective cases".

*Assay for specific glucocorticoid-binding sites.*—Viable cells from the peripheral blood of patients with leukaemia were used at concentrations between 4 and $6\times10^6$ cells/ml for the receptor assay. The receptor assay was carried out by slightly modifying the procedure reported by Lippman *et al.* (1977).

Dexamethasone dissolved in ethanol was further diluted in RPMI-1640 medium (10% foetal calf serum). The final concentration of ethanol to dissolve dexamethasone ($<0{\cdot}1\%$) did not affect cell viability. Each cell suspension (0·8 ml) in RPMI-1640 medium (10% foetal calf serum) was distributed into small glass tubes. To half of these tubes, 0·2 ml of a 5-fold concentration of $^3$H-dexamethasone was added. To the other half, 0·2 ml of a 5-fold concentration of $^3$H-dexamethasone plus a 100-fold excess of unlabelled dexamethasone was added. The binding curves were made by using varying concentrations of $^3$H-dexamethasone with or without unlabelled dexamethasone. Incubations were performed for 2 h at 21°C. Every 15 min, the cells were gently mixed. After incubation, the cell suspensions were washed $\times3$ with cold PBS, and the cells were solubilized in 0·5 ml of Soluene 350 (Packard). Each solubilized sample was transferred to liquid-scintillation vials, and counted in 10 ml of Dimilume-30 (Packard). The viability of the incubated cells was $>96\%$, as examined by using trypan-blue dye exclusion. Binding sites per cell and equilibrium dissociation constants were calculated from Scatchard analysis (Scatchard, 1949) assuming that each receptor has only one steroid-binding site. Maximum specific binding at infinite steroid concentrations was obtained from the "x" intercept of the Scatchard plot. The amount of binding, when converted to the number of molecules bound, divided by the cell number used for the assay, yields the number of specific glucocorticoid binding sites/cell.

*Preparation of cell extract and enzyme assay.*—This was done as described previously (Sasaki *et al.*, 1979). Leukaemic cells separated from the marrow aspirates of fresh leukaemia cases were suspended at $10^8$ cells/ml in extraction buffer (50mM Tris-HCl, pH 7·6), disrupted by freezing and thawing with the addition of 0·25% Triton X-100, and centrifuged at 100,000 *g* for 1 h. The reaction mixture contained 50mM Tris-HCl at pH 7·5, 30mM KCl, 0·5mM $MnCl_2$, 2mM dithiothreitol, 10 $\mu$g of bovine serum albumin, 0·5 u of poly-dA, 0·25mM [$^3$H]-dGTP and the cell extract. Incubations were for 20 min at 37°C. Aliquots were placed on Whatman GF/C filters, and processed according to the method of Chang & Bollum (1971). The samples were counted in a toluene scintillator using a liquid-scintillation spectrometer. Endogenous radioactivity (no primer) was subtracted from the activity of the poly-dA-primed reaction. The enzyme activity was expressed as u/$10^8$ nucleated cells. One unit represents 1 nmol of [$^3$H]-dGMP polymerized on to poly-dA for 20 min at 37°C.

*Immunofluorescence.*—The percentage of TdT$^+$ cells was surveyed by using the indirect immunofluorescence method, as reported previously (Sasaki *et al.*, 1980). Slides were fixed with methanol at 4°C, incubated with rabbit antisera against calf-thymus TdT, and stained with fluoresceinated goat anti-rabbit IgG $(Fab')_2$ fragments. Cells with fluorescent nuclei were scored as positive. This antibody shows good reaction with human terminal transferase (Bollum, 1975, 1979).

*Statistics.*—$\chi^2$ test, unpaired *t* test or evaluation of correlation coefficient, were carried out for statistical analysis of the results (Snedecor & Cochran, 1967).

### RESULTS

Table I summarizes the effect of VP therapy on TdT+ or TdT− leukaemias. Twenty-two (66·7%) of 33 patients with TdT+ leukaemia entered into complete remission after VP therapy. Seven (21·2%) of them showed a favourable response to VP therapy ("effective" cases) though they did not enter into complete remission. Four patients (12·1%) with TdT+ leukaemia showed a poor response to this therapy ("ineffective" cases). However, only 1 (8·33%) of 12 patients with TdT− leukaemia entered into complete remission, and 4 more (33·3%) showed a favourable response ("effective" cases).

TABLE I.—*The effect of vincristine–prednisolone therapy on leukaemia cases*

| | TdT | No. of cases | Response to VP therapy CR | Eff. | Ineff. |
|---|---|---|---|---|---|
| ALL | + | 19 | 14 | 3 | 2 |
| | − | 1 | 0 | 0 | 1 |
| CML in BC | + | 14 | 8 | 4 | 2 |
| | − | 11 | 1 | 4 | 6 |

VP therapy was tried as the first choice for each patient with ALL or CML in blast crisis, and the response was estimated for each case as CR (complete remission) Eff. (effective) and Ineff. (ineffective) (see Materials & Methods for definitions). The presence of TdT was determined by immunofluorescent analysis.

In Table I, the $\chi^2$ test showed a significant difference ($P < 0{\cdot}01$) between TdT+ and TdT− leukaemias in the effect of VP therapy.

Table II shows TdT activity, immunofluorescence analysis, the frequency of GC receptor sites and the effect of VP therapy in patients with ALL or CML in blast crisis. In this Table, the $t$ test also showed a significant difference ($P < 0{\cdot}02$) between VP effective and ineffective cases in level of TdT activity. As shown in Table II, 5/6 TdT+ leukaemias showed a moderate to high level of GC receptors whereas, in ALL or CML in blast crisis with no TdT activity, the level of GC receptors was low; in other words, ALL Cases 1–3 and Case 6 (non-T, non-B ALL) and CML Case 3 ($Ph^1$-positive CML in lymphoblastic crisis, null-cell type) showed high levels of TdT and GC receptors, and responded well to VP. In ALL Case 6, VP induced disappearance of blasts from peripheral blood and decreased hepatosplenomegaly. However, blasts (27·6% of nucleated cells) still remained in this patient's marrow after this therapy. Similarly, in CML Case 3, blasts (32·8% of nucleated cells) still remained in the marrow, although marked cytoreduction, complete elimination of the blasts from peripheral blood and disappearance of splenomegaly were induced by this therapy.

Blasts in ALL Case 4, CML Case 1, 2 (CML in myeloblastic crisis) and 4 contained low levels of GC receptor and no TdT activity. These cases showed poor

TABLE II.—*TdT activities, immunofluorescent analysis, GC receptor sites and the response to VP therapy in leukaemias*

| Case | | Age (yrs) | TdT U/10⁸ | % TdT+ cells | GC receptor Sites/cell | Kd(M × 10⁻⁹) | Response to VP therapy |
|---|---|---|---|---|---|---|---|
| ALL | 1 | 17 | 0·431 | 69·6 | 52242 | 8·81 | CR |
| | 2 | 4 | 0·316 | 68·2 | 17450 | 4·33 | CR |
| | 3 | 53 | 0·199 | 50·5 | 22036 | 12·82 | CR |
| | 4 | 57 | 0·016 | 0·4 | 5371 | 6·89 | ineff. |
| | 5 | 28 | 0·201 | 32·3 | 8652 | 12·20 | ineff. |
| | 6 | 24 | 0·603 | 52·1 | 26284 | 4·02 | eff. |
| CML in BC | 1 | 46 | 0·004 | 0·4 | 2130 | 11·76 | ineff. |
| | 2 | 56 | 0·049 | 0·8 | 8146 | 8·19 | ineff. |
| | 3 | 49 | 0·693 | 63·1 | 42363 | 3·95 | eff. |
| | 4 | 45 | 0·055 | 0·7 | 8504 | 7·77 | ineff. |

In the biochemical assay, TdT− samples contained < 0·06 u/10⁸ cells. Marrow slides from TdT− leukaemias contained < 1% positive cells on immunofluorescence analysis.

Kd(M) = dissociation constant (molarity).

response to VP therapy. ALL Case 5 was also diagnosed as non-T, non-B ALL. However, blasts from this case contained a low level of GC receptor, and showed poor response to VP.

The figure shows the correlation between TdT activity and level of GC receptor sites of the leukaemias shown in Table II. In these cases significant correlation

FIGURE.—The correlation between the level of TdT activity and the number of glucocorticoid receptor sites in ALL or CML in blast crisis. ●, ALL responding to VP; ○, ALL, VP-ineffective; ■, CML responding to VP; □, CML, VP-ineffective.

($r = 0{\cdot}821$, $P < 0{\cdot}01$) was observed between the enzyme activity and the receptor level. Also, in Table II, there was a significant difference ($P < 0{\cdot}01$) between VP effective and ineffective leukaemias in the number of GC receptor sites by unpaired *t* test.

### DISCUSSION

It is well known that in normal tissues of adult animals a major population of $TdT^+$ cells is localized in thymic cortex, and a minor population is in certain lymphoid cells of the marrow (Bollum, 1979). At the infantile stage of rats and mice, another $TdT^+$ cell population appears in peripheral blood, liver, spleen and lung (Bollum, 1979; Sasaki *et al.*, 1980). Treatment of rats with dexamethasone rapidly induces complete elimination of $TdT^+$ cells from all these tissues (Gregoire *et al.*, 1979; Sasaki *et al.*, 1980). Therefore, $TdT^+$ cells in normal tissues are markedly sensitive to glucocorticoid hormone. The unique tissue distribution (Bollum, 1979), glucocorticoid sensitivity and cytochemical characteristics of $TdT^+$ cells (Sasaki & Bollum, in preparation) strongly supports the idea that they are immature precursors of lymphoid cells.

In leukaemia, glucocorticoid hormone has been known as a very effective drug for the therapy of ALL patients, $> 90\%$ of whom show raised levels of TdT. Furthermore, VP therapy has recently been suggested as effective on the treatment of $TdT^+$ leukaemias (Gordon *et al.*, 1978; Marks *et al.*, 1978; Pangalis & Beutler, 1979; Sasaki *et al.*, 1979).

The finding shown in Table I also supports these previous reports. Therefore, blasts from $TdT^+$ leukaemias may retain glucocorticoid sensitivity like normal $TdT^+$ cells, even after their leukaemic transformation. However, the mechanism of the action of glucocorticoid hormone on $TdT^+$ leukaemic cells still remains to be elucidated.

In the leukaemia cases shown in Table II, there was a significant correlation between the level of TdT activity and the number of GC receptor sites. Although we can not draw a definite conclusion from this preliminary result, the presence of high or moderate levels of GC receptor sites in blasts of $TdT^+$ leukaemias suggests that GC receptor may be responsible for the effect of VP therapy on $TdT^+$ leukaemias.

However, there are also some $TdT^+$ leukaemias which fail to respond to VP. $TdT^+$ ALL (Case 5) was markedly resistant to VP, and blasts in this case contained a low level of GC receptors. In this case, the heterogeneity of blasts might be responsible for the low level of GC receptors and poor response to VP. Alternatively, the level of GC receptor sites in blasts might be more important than the level of TdT activity in predicting the response of leukaemic cases to VP therapy. Further analysis of more leukaemias into the relation between TdT activity, GC receptors and the effect of VP therapy is now in progress.

The technical assistance of Miss T. Kumakura is gratefully acknowledged. This work was supported by a grant in aid for cancer research from the Ministry of Health and Welfare.

### REFERENCES

Bollum, F. J. (1975) Antibody to terminal deoxynucleotidyl transferase. *Proc. Natl Acad. Sci., U.S.A.*, **72**, 4119.

Bollum, F. J. (1979) Terminal deoxynucleotidyl transferase as a hematopoietic cell marker. *Blood*, **54**, 1203.

Chang, L. M. S. & Bollum, F. J. (1971) Low molecular weight deoxyribonucleic acid polymerase in mammalian cells. *J. Biol. Chem.*, **246**, 5835.

Coleman, M. S., Greenwood, M. F., Hutton, J. J., Bollum, F. J., Lampkin, B. & Holland, P. (1976) Serial observations on terminal deoxynucleotidyl transferase activity and lymphoblast surface markers in acute lymphoblastic leukemia. *Cancer Res.*, **36**, 120.

Gordon, D. S., Hutton, J. J., Smalley, R. V., Meyer, L. M. & Vogler, W. R. (1978) Terminal deoxynucleotidyl transferase (TdT), cytochemistry and membrane receptors in adult acute leukemia. *Blood*, **52**, 1079.

Greenwood, M. F., Coleman, M. S., Hutton, J. J. & 4 others (1977) Terminal deoxynucleotidyl transferase distribution in neoplastic and hematopoietic cells. *J. Clin. Invest.*, **59**, 889.

Gregoire, K. E., Goldschneider, I., Barton, R. W. & Bollum, F. J. (1979) Ontogeny of terminal deoxynucleotidyl transferase positive cell in lymphohemopoietic tissues of rat and mouse. *J. Immunol.*, **123**, 1347.

Lippman, M. E., Perry, S. & Thompson, E. B. (1977) Cytoplasmic glucocorticoid binding proteins in glucocorticoid-unresponsive human and mouse leukemic cell lines. *Cancer Res.*, **34**, 1572.

Marks, S. M., Baltimore, D. & McCaffrey, R. (1978) Terminal transferase as a predictor of initial responsiveness to vincristine and prednisolone in blastic chronic myelogenous leukemia. *N. Engl. J. Med.*, **298**, 812.

McCaffrey, R., Smoler, D. F. & Baltimore, D. (1973) Terminal deoxynucleotidyl transferase in a case of childhood acute lymphoblastic leukemia. *Proc. Natl Acad. Sci., U.S.A.*, **70**, 521.

Pangalis, G. A. & Beutler, E. (1979) Terminal transferase in leukemia of adults. *Acta Haematol.*, **62**, 199.

Sarin, P. S., Anderson, P. N. & Gallo, R. C. (1976) Terminal deoxynucleotidyl transferase activities in human blood leukocytes and lymphoblast cell lines. High levels in lymphoblastic cell lines and in blast cells of some patients with chronic myelogenous leukemia in acute phase. *Blood*, **47**, 11.

Sasaki, R., Bollum, F. J. & Goldschneider, I. (1980) Transient populations of terminal transferase positive ($TdT^+$) cells in juvenile rats and mice. *J. Immunol.*, **125**, 2501.

Sasaki, R. & Sakamoto, S. (1977) The function and clinical significance of DNA polymerases in human hematopoietic cells. *Acta Haematol. Jpn*, **40**, 252.

Sasaki, R., Takaku, F., Sakamoto, S. & Kanoh, Y. (1979) Terminal deoxynucleotidyl transferase activity and B cell markers in chronic myelogenous leukemia blast crisis. *Acta Haematol. (Basel)*, **62**, 143.

Scatchard, G. (1949) The attractions of proteins for small molecules and ions. *Ann. N.Y. Acad. Sci.*, **51**, 660.

Snedecor, G. W. & Cochran, W. G. (1967) *Statistical Methods*. Ames, Iowa: Iowa State University Press. pp. 20 & 59.

Stass, S. A., Schumacher, H. R. Keneklis, T. P. & Bollum, F. J. (1979) Terminal deoxynucleotidyl transferase immunofluorescence of bone marrow smears: Experience in 156 cases. *Am. J. Clin. Pathol.*, **72**, 898.